# Effectiveness of Ultra-Low Volume Nighttime Applications of an Adulticide against Diurnal *Aedes albopictus*, a Critical Vector of Dengue and Chikungunya Viruses

**DOI:** 10.1371/journal.pone.0049181

**Published:** 2012-11-08

**Authors:** Ary Farajollahi, Sean P. Healy, Isik Unlu, Randy Gaugler, Dina M. Fonseca

**Affiliations:** 1 Department of Entomology, Center for Vector Biology, Rutgers University, New Brunswick, New Jersey, United States of America; 2 Mercer County Mosquito Control, West Trenton, New Jersey, United States of America; 3 Monmouth County Mosquito Extermination Commission, Eatontown, New Jersey, United States of America; Johns Hopkins University, United States of America

## Abstract

*Aedes albopictus*, the Asian tiger mosquito, continues expanding its geographic range and involvement in mosquito-borne diseases such as chikungunya and dengue. Vector control programs rarely attempt to suppress this diurnal species with an ultra-low volume (ULV) adulticide because for maximum efficacy applications are conducted at night. During 2009–2011 we performed experimental nighttime applications of a novel adulticide (DUET®) against field populations of *Ae. albopictus* within an urban site composed of approximately 1,000 parcels (home and yard) in northeastern USA. Dual applications at mid label rate of the adulticide spaced one or two days apart accomplished significantly higher control (85.0±5.4% average reduction) than single full rate applications (73.0±5.4%). Our results demonstrate that nighttime ULV adulticiding is effective in reducing *Ae. albopictus* abundance and highlight its potential for use as part of integrated pest management programs and during disease epidemics when reducing human illness is of paramount importance.

## Introduction

Chikungunya fever is an emerging tropical mosquito-borne disease caused by the chikungunya virus (CHIKV, genus *Alphavirus*, family *Togaviridae*) that has become widespread in the Indian Ocean region, resulting in millions of disease cases and over 250 deaths [1]. While the acute febrile phase of the disease is usually resolved in a few days, the associated joint pain may persist indefinitely; further causing health and economic impact [2]. Although historically not an important vector of CHIKV, the Asian tiger mosquito, *Aedes albopictus* (Skuse) has recently emerged as the principal driver of epidemics of chikungunya [3] after a single amino acid mutation in the envelope protein of CHIKV increased its vector competence [4], [5].

Due to the widespread and increasing distribution of *Ae. albopictus* in temperate regions of North America and Europe [6], [7], [8] and the escalating diagnoses of cases in travelers returning from endemic or epidemic areas [9], [10] the risk of local CHIKV transmission in these continents is no longer conjectural, as revealed by an epidemic comprising over 200 autochthonous cases in Italy during 2007 [11] as well as sporadic autochthonous cases in France [3]. Due to the absence of a vaccine for CHIKV, mosquito control, particularly the reduction of biting populations of the primary vector *Ae. albopictus*, is the only effective means of reducing chikungunya fever cases during an epidemic.

Most federal and state guidelines for protecting the public during outbreaks of mosquito-borne diseases recommend adulticides from aircraft and truck-mounted equipment as the most effective method of reducing transmission risk to humans [12]. These adulticide interventions are generally applied as ultra-low volume (ULV) cold aerosol sprays during night-time campaigns when a thermal inversion has occurred to keep the insecticide from dispersing upwards and light winds aid in the spread of the insecticide droplets [13]. Because prior ULV applications have not been efficacious or long lasting in controlling diurnally active urban mosquitoes, such as *Aedes aegypti* (L.) [14], [15] and *Ae. albopictus* [16], they have been declared ineffective in reducing dengue transmission [17]. One reason for failure of control may be the nocturnal resting behavior of day-biting mosquitoes in natural and artificial places that are sheltered from the insecticide plume [18]. The ineffectiveness of nighttime ULV applications against diurnal mosquitoes has become the conventional wisdom within the modern vector control community in the USA and many mosquito abatement programs simply do not attempt to adulticide against *Ae. albopictus* (D. Ninivaggi, personal communication). Since the public health implications of an *Ae. albopictus*-driven arboviral epidemic are great, vector control officials must be adequately prepared to intervene with efficacious application strategies and products. A critical need exists for novel methods of insecticide application or new formulations to achieve successful control.

DUET™ Dual-action Adulticide (Clarke®, Roselle, IL, USA) is a new commercially available adulticide for mosquito control that causes a benign agitation [a non-biting excitation of mosquitoes] potentially flushing mosquitoes from resting places and increasing contact with airborne droplets that are more likely to impinge on flying adults [19]. DUET adulticide combines the pyrethroids sumithrin (5%, 44.94 g/L Active Ingredient) and prallethrin (1%, 8.99 g/L AI) with the synergist piperonyl butoxide (5%, 44.94 g/L AI). Prallethrin is reported to induce an excitatory response at sublethal concentrations and may drive mosquitoes from a resting state and expose them to lethal doses of airborne sumithrin and piperonyl butoxide [19]. This adulticide may have advantages against not only resting gravid or engorged mosquitoes but also against diurnal mosquitoes such as *Ae. albopictus* which may be inactive during routine nighttime ULV applications by mosquito abatement programs.

The objective of this study was to evaluate the area-wide efficacy of nighttime (01∶00–06∶00) ground-applied ULV adulticide applications of DUET against *Ae. albopictus* within an urban residential community; we compared the abundance of *Ae. albopictus* populations within treated and untreated areas of Mercer County, New Jersey during 2009–2011. Our ultimate goal was to develop a successful ULV adulticide application strategy to be used in an integrated pest management (IPM) program for suppression of *Ae. albopictus*, both for nuisance reduction and to address imminent future outbreaks of chikungunya and dengue fever.

## Materials and Methods

### Study Area

**Figure 1:**
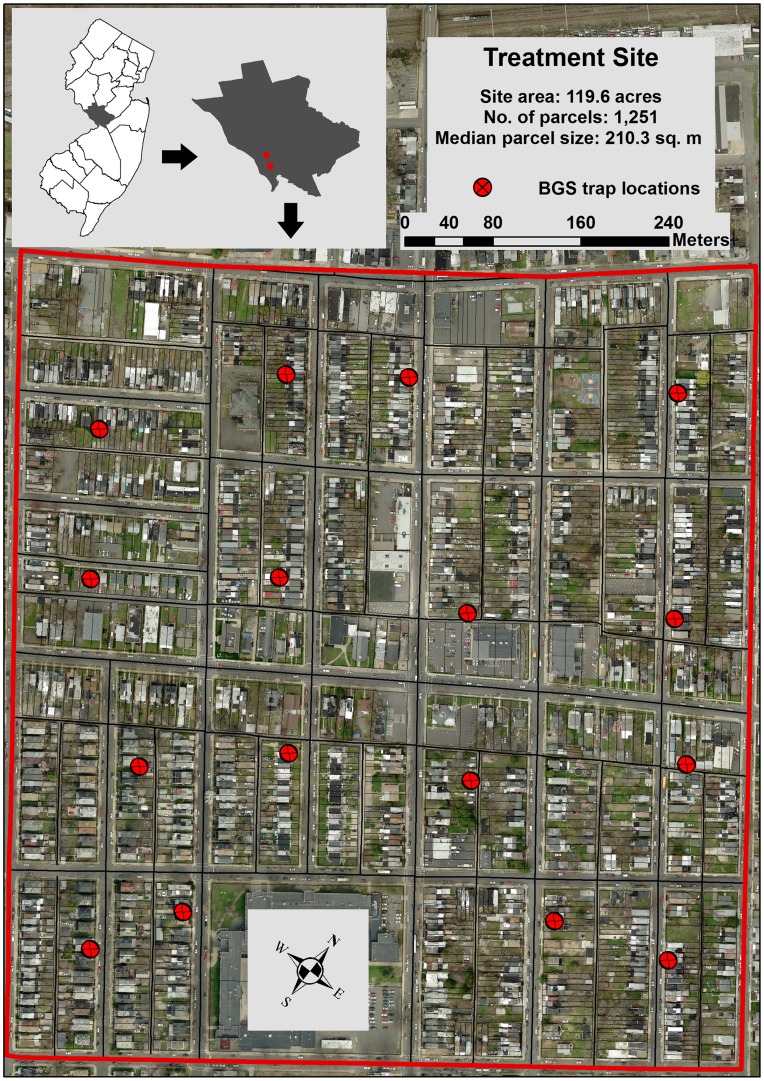
Map of ULV adulticide treatment site in Mercer County, New Jersey, USA, 2009–2011. Inset of Mercer County in the top left displays locations of treatment and no intervention sites, and detailed map below displays locations of BGS traps, parcels, and roads/alleys within only the treatment site. A typical block within this highly urbanized location is about 90 m wide and 150 m long, with each block divided by a drivable alley behind each parcel. All roadways and alleys were driven during an adulticide application.

During 2009, a highly urbanized residential field site was chosen in Mercer County, New Jersey, USA (40° 13′ N, 74° 44′ W) as part of an area-wide management of the Asian tiger mosquito [20]. The field site (Treatment Site) is located within the City of Trenton (population ∼ 83,000, area 21.1 km^2^) and consists of 48.4 ha, including 1,251 parcels (Figure 1). Parcels correspond to a structure or house with surrounding yard, and are most often built as adjoining row homes or duplexes, indicative of the type of housing in this area. Almost all adjoining parcels contain a sheltered alcove area between two homes, where vegetation and trash proliferate, affording mosquitoes a shaded and humid area for a resting place. Additionally, socioeconomic conditions within the field site have led to a large number of abandoned homes that have been boarded shut by the City of Trenton, but often house transient humans and large amounts of trash [20]. Lack of ownership and responsibility for hygiene has increased mosquito populations within these parcels. Our field site consists of roughly 26 residential blocks, each containing a residential street on all four sides, and divided between parallel parcels by a drivable alley. During ULV adulticide applications, streets and alleys are both driven to maximize dispersal of insecticide. A second field site (40° 12′ N, 74° 43′ W), similar in both socioeconomic conditions and *Ae. albopictus* levels [20], was chosen as an untreated control (Control Site), where no active interventions were performed against *Ae. albopictus*. This site consisted of 62.4 ha, including 1,064 parcels and was solely used to sample adult mosquito populations using the same protocol used in the treatment site [21].

### Ultra-low Volume Adulticide Application

A Cougar® (Clarke Mosquito Control, Roselle, IL, USA) cold aerosol ULV generator was used during all adulticide applications. The unit was fitted with a SmartFlow (Clarke Mosquito Control, Roselle, IL, USA) system used in tandem with radar ground speed of the vehicle to ensure appropriate flow of insecticide and accurate reporting and tracking of amount of chemical used along with distance and area sprayed (Mention of trade names or commercial products are solely for the purposes of providing specific information and do not imply endorsement by the authors or other involved parties). The sprayer was mounted in the back of a flatbed truck at a height of 1.8 m, and the spray boom was angled 45.5° pointing backwards. The vehicle was driven at an average speed of 16.1 km/h. Droplet size measurements were obtained for the Cougar ULV machine prior to operational applications using a DC-III portable droplet measurement system (KLD Laboratories, Huntington Station, NY, USA). For vector spraying a droplet size range of 5 to 25 µm is most efficient, because this size is most likely to impinge on a mosquito and deliver a toxic dose [22].

Droplet measurements for mosquito control are often provided as a mass median diameter or a volume median diameter (VMD). The VMD is also routinely provided as DV_0.5_, a term used to represent a statistic where 50% of the spray volume or mass is contained in droplets smaller than this value. Most often, values for a DV_0.1_ and a DV_0.9_ are also provided, to describe 10% and 90% of the cloud volume, respectively. Droplet size and distribution are two of the most important factors affecting the success of an ULV application [23]. Additionally, adulticide labels, which are interpreted as federal law, require that given equipment adhere explicitly to required VMD values. We conducted two readings using the DC-III during our calibration of the Cougar ULV sprayer and acquired a DV_0.1_ value of 2.9 µm, a VMD (DV_0.5_) value of 15.2 µm, and a DV_0.9_ value of 30.8 µm. A total of 4,015 drops were counted, with only 6 droplets above 32 µm in size, and none above 48 µm.

The pesticide label for DUET requires ground-based spray equipment to be adjusted to deliver aerosolized droplets within a VMD of 8 to 30 µm (DV_0.5_<30 µm) and a DV_0.9_ value of less than 50 µm. For all field trials, DUET was applied at a flow rate of 136.04 ml/min. Applications during 2009 were conducted at maximum allowable label rate for a ground ULV spray (86.2 g/ha). This full label rate results in 0.81 g/ha AI of prallethrin, 4.04 g/ha AI of sumithrin, and 4.04 g/ha AI of piperonyl butoxide. Subsequent applications during 2010–2011 were conducted at recommended mid label rate (42.7 g/ha), resulting in 0.40 g/ha AI of prallethrin, 2.02 g/ha AI of sumithrin, and 2.02 g/ha AI of piperonyl butoxide. Only single adulticide applications were conducted during 2009, however, in order to increase efficacy by compensating for gaps in coverage and missed targets, we conducted dual applications of the adulticide spaced one or two days apart during 2010 (twice) and 2011 (once). Our intention was to control adult populations with the first ULV application, wait one or two days, and conduct another adulticide application to control any newly emerged adults or mosquitoes that may have been missed with the initial application.

Truck-mounted adulticide applications were conducted at night using a single vehicle to drive the entire treatment site. Routes were designed to follow all available roads and alleys to provide maximum coverage. Each application took about 2 hours to complete and was conducted between 01∶30–06∶30, depending on the date of the application.

### Adult Mosquito Surveillance and Analysis

Mosquitoes were sampled in our treatment site and control site on a weekly basis during 2009–2011 utilizing a network of BioGents Sentinel™ (BGS) traps (Biogents AG, Regensburg, Germany). Specific details of surveillance protocols are outlined elsewhere [21]; but briefly, locations were chosen by overlaying a grid of specific distance intervals. We used a 175–200 m distance between BGS traps for each site. Locations were determined using the Fishnet tool within ArcGIS Desktop 9.2 (Environmental Systems Research Institute, Redlands, CA, USA). These distances were based on current knowledge of *Ae. albopictus* flight range [24] and the available resources within each site. Two hundred meter sampling resulted in 12 traps within the treatment site and 15 traps within the control site during 2009–2010, while 175 meter sampling resulted in 16 traps within the treatment site and 24 traps within the control site during 2011. Sampling was performed with BGS traps deployed weekly for 24 hours and deployed in backyards (near vegetation or shade) of each parcel selected. Each week, traps were placed in the same location within the backyards. Permissions to place BGS traps within each parcel were acquired at the beginning of each season from individual property owners. The BGS trap was used with a solid BG-lure (Biogents AG, Regensburg, Germany) containing ammonia, lactic acid and fatty acids, components known to be particularly attractive to *Ae. albopictus* [25].

Mosquitoes recovered from traps were placed in containers and transported to the laboratory on dry ice for identification and pooling. We calculated the mean number of *Ae. albopictus* adults (male+female) collected during each sampling session in BGS traps within each site. Adulticide applications were performed when environmentally, logistically, and operationally feasible within the treatment site when a threshold mean of ≥5 *Ae. albopictus* (male+female) adults were detected in our weekly BGS surveillance. This number was chosen because 3 bites have been reported as a common nuisance threshold driving residents indoors [26], and an average of 5 bites/day by *Ae. albopictus* in Italy has been recorded as intolerable [27]. Percent control after ULV application of adulticides was calculated by using an algebraic variation of Henderson’s method [28] using the formula: percent control = 100–[(T/U)100], where T is the post application mean divided by the pre application mean in the treatment site and U is the post application mean divided by the pre application mean in the control (no intervention) site. Although additional integrated pest management intervention efforts such as education, source reduction, and application of larvicides were being conducted within our treatment site as part of a larger project [21], none would have an immediate effect on adult populations. Thus, our analyses concentrated on the overnight percent reduction of adult populations. We used ANOVA (JMP 8, SAS Institute, Cary, NC, USA) to examine the efficacy of a single ULV application versus a dual application, and full label rate versus mid label rate. Percentages were arcsin transformed prior to analysis [29]. No specific permits were required for the collection of adult mosquitoes or the described field studies, which were developed with homeowners assent by professional county mosquito control personnel. These studies did not involve endangered or protected species.

### Meteorological Data Collection

During each application, meteorological data was recorded for wind speed, direction, humidity, and temperature at 1 m and 10 m heights for thermal inversion observation. A Vantage Pro2 (Davis Instruments, Hayward, CA, USA) portable weather station was set up within the treatment site 2 hours prior to application and maintained until 2 hours post application. Additional meteorological data was obtained from a permanent weather station located at Trenton-Mercer Airport, situated 7.5 km from the application site.

## Results

The experiments were performed during the 2009, 2010, and 2011 active seasons for *Ae. albopictus*. Adulticide applications were conducted in unison with an intensive surveillance program and were one of the components of an IPM strategy being developed for control of *Ae. albopictus*. We conducted our first application of DUET at full label rate and then proceeded to evaluate mid label rate applications in different combinations (Table 1). Although most applications of adulticide were initiated when the mean number of adults (male+female) captured in BGS traps were above 5, on one occasion we started with lower numbers (4.1±1.4) because we were testing the effect of adulticiding on populations of *Ae. albopictus* at the end of the season. Although evaluating the efficacy of control measures may be more difficult when adult numbers are already low, this test yielded control levels similar to those at other mid label rate single applications (Table 1). As a result, the removal of this treatment from the analysis does not affect the overall results (data not shown). The number of post-treatment adults was measured for 24 hrs starting the afternoon of the day (night) when treatment occurred. For duplicate treatments, the post-treatment counts were made after the second treatment only. In all cases post-treatment values were lower than 5 (2.3±0.7). The absence of significant wind was a constant (Table 1) as well as high humidity and air temperatures at night in the mid 20°C range, which are characteristic of urban areas in mid Atlantic states during the summer months [30].

**Table 1:** Summary of adulticide applications and BGS trap results during 2009–2011, Mercer County, New Jersey.

Year	Application Date	ApplicationTime (am)	ApplicationRate (gm/ha)	Temp (°C)	Relative Humidity(RH %)	Wind Speed(km/h)	Treatment Site		Control Site		Percent Control*
							Pre-treatment Mean (±SE)	Post-treatment Mean (±SE)	Pre-treatment Mean (±SE)	Post-treatment Mean (±SE)	
2009	05-Aug-09	02:45 to 05:00	86.2	22.2	94%	<1.6†	8.3±2.0	2.0±0.7	27.1±5.3	16.5±2.8	61%
	19-Aug-09	03:15 to 06:00	86.2	22.8	93%	5.6	14.3±4.0	4.1±0.9	18.1±4.7	21.4±5.0	74%
	16-Sep-09	03:00 to 05:00	86.2	19.2	76%	10.5	7.8±2.1	1.1±0.4	19.7±3.9	16.3±3.8	83%
2010	29-Jul-10 &30-Jul-10‡	04:00 to 05:30 &04:00 to 06:00	42.7	25.3 & 18.9	85% & 65%	8.1 & 9.9	10.8±2.9	2.6±0.7	9.1±1.7	13.9±3.6	82%
	18-Aug-10 &20-Aug-10‡	05:00 to 06:30 &05:30 to 07:00	42.7	22.2 & 21.1	69% & 73%	<1.6 & <1.6	10.5±2.3	0.9±0.3	14.2±2.8	11.4±1.4	90%
	02-Sep-10	05:00 to 06:00	42.7	22.8	73%	<1.6	5.7±1.2	3.1±1.0	12.1±2.3	11.5±1.7	43%
2011	4-Aug-11 &5-Aug-11‡	01:45 to 03:25 &03:30 to 05:00	42.7	20.6 & 22.2	87% & 95%	5.6 & <1.6	6.6±1.2	1.6±0.6	10.3±1.6	14.9±1.8	83%
	14-Sep-11	02:30 to 05:00	42.7	21.1	87%	3.2	15.5±2.8	3.2±1.3	26.3±5.0	17.3±3.4	68%
	27-Sep-11	02:30 to 04:00	42.7	21.7	93%	<1.6	4.1±1.4	2.5±0.7	12.7±2.8	16.4±4.0	54%

* Percent control following Henderson’s equation: 100–[(T/U)*100].

† Wind speeds of <1.6 km/h indicate that wind was negligible during ULV application.

‡ Denotes a tandem application of adulticide.

We found that single ULV adulticide applications at the full label rate of 86.2 gm/ha resulted in a percent reduction of 72.7±5.4% (SE), which is significantly higher [*p* = 0.04] than single ULV applications at the mid label rate of 42.7 gm/ha (54.0±4.7%). However, dual applications at mid label rate were significantly more effective (*p* = 0.003) than single applications at full rate and resulted in an average percent reduction of 85.0±5.4%. Dual applications at the full label rate could not be conducted without exceeding label guidelines. Overall the two variables, application rate (full versus mid) and application type (single versus dual), explained 75% of the variance in percent control (R^2^ = 0.75).

## Discussion

Evaluating the efficacy of aerosol sprays for adult mosquito control is critical to assessing their suitability, especially during epidemics when fast reduction in populations of biting females is paramount. Over three years and multiple nighttime adulticide applications, we observed an overall significant average percent reduction in adult populations of day-biting *Ae. albopictus* mosquitoes as measured using BGS trap surveillance. Our results provide direct evidence that nighttime applications of an ULV adulticide are effective in reducing *Ae. albopictus* abundance.

Our measures of adult population reductions were derived from BGS traps, a relatively new sampling device for capturing container-inhabiting *Aedes* mosquitoes. The BGS trap has been proven as an effective alternative to other collection devices and traps such as backpack aspirators, gravid traps, variations of carbon dioxide-baited traps, and the Fay-Price trap [25], [31], [32] for obtaining estimates of field abundance of *Ae. albopictus*, and approximates human landing rate estimates [32], [33]. By targeting adult mosquitoes, BGS traps provide an actual estimate of the biting populations, and hold an immediate advantage over other sampling and population assessment methods (e.g. Breteau, container, house indices or pupae per person) which are relatively more labor intensive and plagued with levels of assumptions, imprecision, and unpredictability [34]. BGS traps provide an opportunity for improved adult entomological surveillance and have been used successfully as a rapid response tool for detection of *Ae. albopictus* [35] and to gauge efficacy of various control measures targeted against this species [21]. Furthermore, we utilized not only before/after numbers, but also comparisons between treated and untreated sites to determine the immediate percent reduction effects of adulticide applications on populations of *Ae. albopictus* in temperate North America.

Significantly, we found a greater effect on adult *Ae. albopictus* populations through utilization of dual or repeated applications of adulticide at mid label rate. Previous studies have indicated that two adulticide treatments using dieldrin (a chlorinated hydrocarbon similar to DDT which is now banned in most of the world) as a thermal fog during the day and spaced a week apart, increased and prolonged control of *Ae. albopictus* for up to eight weeks [36]. Adulticide interventions by aircraft during the day against *Ae. aegypti* using malathion applied twice 4 days apart have also shown upwards of 90% control for over 10 days post application [37]. We conducted dual ULV applications of adulticide at mid label rate resulting in an average reduction of 85% in *Ae. albopictus*. Furthermore, although previous studies have indicated that ULV adulticides need to be applied at maximum rate [13], [38], we found that even mid label rate applications of the insecticide had a significant effect on *Ae. albopictus*. Our field applications were conducted in a highly urbanized area in which we were able to drive both roadways and alleys to further enhance penetration of product and contact with mosquitoes. This finding has promising potential for vector control programs that are often under scrutiny about pesticide costs and also usage/exposure from the general public and must face increasing regulations and adulticide amount limits from local/federal government.

The rationale for adulticiding during epizootics or epidemics of arboviruses is to reduce the number of infected mosquitoes and thus interrupt pathogen transmission. Studies of *Ae. aegypti* following ULV adulticide applications have shown that only 8% of female mosquitoes dissected post-treatment were parous, as compared with parity rates of 30% in the pre-treatment area and 40% in an untreated area [37]. The reduction in parous females, which are most likely to be infected, makes ULV adulticiding a very important component of a comprehensive intervention program geared towards protection of public health from mosquito-borne diseases. Careful examination of the 2007 outbreak of chikungunya fever in Italy, the first large outbreak in a temperate climate region, indicates that a larger epidemic was thwarted by timely control interventions [39]. Although it is still debated what level of reduction in adult populations is necessary and sufficient to prevent disease outbreaks, transmission models developed for *Ae*. *aegypti* and dengue suggest that the degree of suppression required to eliminate summertime spread of the disease may be lower than 83% in some cases but closer to 90% in others [34], [40]. The reduction in *Ae. albopictus* abundance we achieved through nighttime adulticiding (85%) would likely result in a decrease in the number of infective bites received by the human population and would consequently impact the transmission of an arbovirus such as dengue or CHIKV.

In conclusion we provide evidence that a nighttime ULV application of a synthetic pyrethroid is efficacious in reducing the abundance of *Ae. albopictus* in an urban environment and that dual applications using mid label rates, spaced one or two days apart, provide levels of reduction in the adult populations of *Ae. albopictus* in the upper range of which is necessary for interruption of arboviral transmission. The large and growing populations of *Ae. albopictus* in several northeastern urban centers such as Washington (DC), Philadelphia, Trenton, and New York City [6], [8] make a large autochthonous outbreak of an arbovirus such as CHIKV or dengue a clear and present danger. We recommend that nighttime applications of ULV adulticides in areas with large populations of *Ae. albopictus* be considered as part of an integrated mosquito management approach for public health protection.
